# Enhanced habit formation in Tourette patients explained by shortcut modulation in a hierarchical cortico-basal ganglia model

**DOI:** 10.1007/s00429-021-02446-x

**Published:** 2022-02-03

**Authors:** Carolin Scholl, Javier Baladron, Julien Vitay, Fred H. Hamker

**Affiliations:** 1 Max-Planck-School of Cognition, Stephanstraße 1A, 04103 Leipzig, Germany; 2 Chemnitz University of Technology, Straße der Nationen 62, 09107 Chemnitz, Germany

**Keywords:** Tourette syndrome, Goal-directed behavior, Habitual behavior, Basal ganglia, Dopamine

## Abstract

Devaluation protocols reveal that Tourette patients show an increased propensity to habitual behaviors as they continue to respond to devalued outcomes in a cognitive stimulus-response-outcome association task. We use a neuro-computational model of hierarchically organized cortico-basal ganglia-thalamo-cortical loops to shed more light on habit formation and its alteration in Tourette patients. In our model, habitual behavior emerges from cortico-thalamic shortcut connections, where enhanced habit formation can be linked to faster plasticity in the shortcut or to a stronger feedback from the shortcut to the basal ganglia. We explore two major hypotheses of Tourette pathophysiology—local striatal disinhibition and increased dopaminergic modulation of striatal medium spiny neurons—as causes for altered shortcut activation. Both model changes altered shortcut functioning and resulted in higher rates of responses towards devalued outcomes, similar to what is observed in Tourette patients. We recommend future experimental neuroscientific studies to locate shortcuts between cortico-basal ganglia-thalamo-cortical loops in the human brain and study their potential role in health and disease.

## Introduction

Neurological disorders, such as the Gilles de la Tourette Syndrome, can reveal fundamental properties of neural circuits involved in learning habitual responses. Tourette patients are typically known for repeatedly making movements and sounds that are not entirely voluntary. Such tics are sometimes described as responses towards involuntary premonitory sensations or urges that stop upon tic execution (Brandt et al. 2016; Kwak et al. 2003; Leckman et al. 1993). This view inspired the comparison of tics and habits, which are automatic and fast, yet inflexible responses towards stimuli. In an outcome devaluation paradigm, unmedicated adult Tourette patients with tics indeed relied on habitual rather than goal-directed behavior, more so than healthy control subjects (Delorme et al. 2016), but refer to de Wit et al. (2018) regarding a debate about outcome-devaluation and habits in humans.

Cognitive symptoms other than tics have been reported in Tourette syndrome (Brand et al. 2002; Robertson et al. 2002; Eddy and Cavanna 2013; Puts et al. 2015), but barely discussed. However, habit reversal training is a commonly used cognitive-behavioral therapy method which aims to replace tics by alternative responses (Dutta and Cavanna 2013). It relies on the idea that tics share key features with habits and thus, may also have a common neural underpinning (Leckman and Riddle 2000).

The dichotomy between habitual and goal-directed behavior has often been associated with separate cortico-basal ganglia loops (Yin and Knowlton 2006; Redgrave et al. 2010), first described as functionally segregated circuits (Alexander et al. 1986). One motor-related loop traverses via the dorsolateral striatum (putamen), receiving substantial input from the motor, the somatosensory, premotor and supplementary motor cortex (Kunzle 1975, 1977; McGeorge and Faull 1989; Mailly et al. 2008). The dorsolateral striatum in turn projects to the ventrolateral areas of the pallidum and to caudolateral portions of the substantia nigra, which project to the ventrolateral nucleus of the thalamus, and finally project back to the same cortical areas that provide inputs to the dorsolateral striatum, creating an independent closed motor loop (Parent et al. 1983, 1984; Alexander and Crutcher 1990). A so-called associative circuit was localized in the dorsomedial striatum (caudate nucleus), which receives its inputs from the prefrontal cortex (Alexander et al. 1986; Alexander and Crutcher 1990; Middleton and Strick 2002). The dorsomedial striatum then projects to the dorsomedial areas of the pallidum, and further to the ventral anterior thalamic nucleus, that projects back to prefrontal areas. Thalamic efferents to the striatum are further topographically organized (Lanciego et al. 2004; Berendse and Groenewegen 1990). Based on this anatomical separation between loops and motivated by results from lesion studies, it was proposed that the associative loop is involved in goal-directed behavior, while the motor loop is involved in habitual behavior (Yin and Knowlton 2006; Redgrave et al. 2010).

More recent investigations, using magnetic resonance diffusion weighted imaging methods, confirm the existence of a topographical segregation, but also reveal a high degree of overlap (Draganski et al. 2008). Several possible communication mechanisms between loops have been proposed (Groenewegen 2003). Early works suggested that information is integrated through widespread output projections from the striatum (Joel and Weiner 1994). Later hypotheses considered the cortico-thalamic-striatal pathway (McFarland and Haber 2002) or the striato-nigro-striatal network (Yin and Knowlton 2006). Recent data shows a strong overlap of corticostriatal connections (Haber 2016; Groenewegen et al. 2017). In monkeys, the overlap reaches as much as 80% depending on the distance between the cortical sites (Averbeck et al. 2014). Similar results were found in rats (Mailly et al. 2013). Therefore, direct communication between loops seems to be more important than initially expected.

The directed transition of goal-directed behavior into habits suggests a more integrated and hierarchical organization of these circuits (Balleine et al. 2015; Yin 2017; Rusu and Pennartz 2020). Specifically, the habitual system may represent a lower level of the hierarchy than the goal-directed one. Baladron and Hamker (2020) proposed therefore a new account to habit formation: A model composed of multiple, hierarchically organized cortico-basal ganglia loops where habitual responses emerge from cortico-thalamo-cortical shortcut connections that bypass the longer and slower route through multiple cortico-basal ganglia loops. Habitual learning transfers behavioral control from the cortico-basal ganglia loops to cortico-thalamo-cortical shortcut connections. Shortcut connections with enough training may directly connect sensory cortical areas with the thalamus of lower-level loops, resulting in a fast excitation of the premotor cortex and the initiation of action.

In the independent loops approach of habits (Yin and Knowlton 2006; Redgrave et al. 2010), where the associative loop implements goal-directed behavior and the motor loop implements motor habits, an arbitration mechanism is required if the loops vote for different actions. However, a shortcut may integrate both aspects without the need of an additional, external mechanism. The understanding of how such shortcuts may be implemented in the brain requires detailed anatomical insight. Classically, cortex is assumed to have reciprocal connections to distinct thalamic parts (Deschenes et al. 1998). However, recent data shows that reciprocal corticothalamic connections are accompanied by non-reciprocal ones (McFarland and Haber 2002; Haber and Calzavara 2009). Further, the prefrontal cortex has reciprocal connections to two thalamic nuclei, the mediodorsal and ventromedial thalamus (Collins et al. 2018). Corticothalamic projections are typically more widespread than thalamocortical projections (McFarland and Haber 2002; Haber and Calzavara 2009). Furthermore, reciprocity at the cell-to-cell level has not yet been demonstrated (Rockland 2015) and recent large-scale tracer experiments show that cortico-thalamic networks are organized in a shallow hierarchy (Harris et al. 2019).

In rats, a cortical site for cortico-thalamo-cortical shortcut connections may be the infralimbic cortex in the medial prefrontal cortex, as lesions of this area prevent them from learning habits (Killcross and Coutureau 2003); yet when the cortical disruption is applied after the learning of habits, goal-directed behavior reoccurs (Coutureau and Killcross 2003; Smith et al. 2012). The model of Baladron and Hamker (2020) provides a framework to understand the ineffectiveness of outcome devaluation after overtraining in rodents (Smith and Graybiel 2013; Adams 1982): animals keep responding towards devalued outcomes because habitual actions are triggered as direct responses to stimuli via shortcuts, circumventing a careful evaluation of goals.

Here, we aim to investigate whether theoretically grounded changes that simulate the suspected pathophysiology of Tourette syndrome, as described in the next paragraphs, may modulate the effect of shortcut connections and in turn produce the enhanced habit formation observed in the outcome devaluation experiment by Delorme et al. (2016). Specifically, we propose that aberrant activation of cortico-thalamo-cortical shortcut connections may increase the rate of response towards stimuli associated with devalued outcomes, resembling the behavior of Tourette patients in the study.

Initial theories regarding Tourette pathophysiology were explained on basis of the popular pathway model of basal ganglia function, according to which tics may occur due to decreased activity of the subthalamic nucleus, or to a selective dysfunction of striatal neurons projecting to the external pallidum (Albin et al. 1989). Abnormal activity of the striatum may cause multiple foci of inhibition in the internal globus pallidus, which could then be reinforced through synaptic plasticity (Mink 2003). The exact mechanism leading to such an unwanted activation of the striatum is unclear, but candidate hypotheses are disturbed dopamine signaling and abnormal inhibitory circuits in the striatum (Albin and Mink 2006).

According to the disturbed dopamine signaling hypothesis of Tourette syndrome (Singer et al. 1982), tonic dopamine levels may be reduced, while in turn phasic dopamine bursts would be increased (Singer et al. 2002; Wong et al. 2008). Yet, because dopamine reuptake inhibitors are less effective in treating tics as expected, Maia and Conceição (2017, 2018) suggest that both tonic *and* phasic dopamine may be increased in Tourette Syndrome. In this framework, increased phasic dopamine bursts may accelerate tic learning by amplifying long-term potentiation on cortico-striatal projections in the direct pathway and long-term depression on projections in the indirect pathway. Increased tonic levels of dopamine may additionally up- and down-regulate the excitability of D1 and D2 striatal cells, thereby reducing the inhibition through the indirect pathway and promoting tic execution (Maia and Conceição 2017).

A second prominent hypothesis of Tourette pathophysiology involves the feedforward and feedback inhibitory circuits within the striatum. In post-mortem analyses of brains, the caudate nucleus of Tourette patients was found to have smaller volume compared to healthy control subjects (Peterson et al. 1998, 2003), a difference which has been linked to a loss of inhibitory interneurons (Kalanithi et al. 2005; Kataoka et al. 2010). A reduced number of interneurons may give rise to local disinhibition within the striatum (Assous and Tepper 2019), which, as a final consequence, would decrease the tonic inhibition of the thalamus and release tics. Animal models of tics seem to support this hypothesis: Tic-like movements after striatal disinhibition have been observed in mice and primates (McCairn et al. 2009; Pogorelov et al. 2015). In rats, experimentally induced acute and chronic striatal disinhibition led to acute and chronic tics, respectively (Bronfeld and Bar-Gad 2013; Vinner et al. 2017).

Based on these hypotheses, we built a spectrum of neuro-computational models that are used to simulate the task-related behavior of control subjects and Tourette patients reported by Delorme et al. (2016). In this experimental study, unmedicated Tourette patients responded more frequently towards stimuli associated with devalued outcomes, indicative of enhanced habit formation, which may relate to tic formation in Tourette syndrome. We successfully replicated this group difference in our simulations by comparing the task performance among different pathological models and a healthy control model whose parameters have been fit to the control group. Particularly, we show that aberrant shortcut activation can make the model rely more on habitual behavior. In line with two major Tourette hypotheses, such aberrant patterns could be indirectly produced by enhanced dopamine modulation or reduced local striatal inhibition.

## Materials and methods

### Task description

**Fig. 1:**
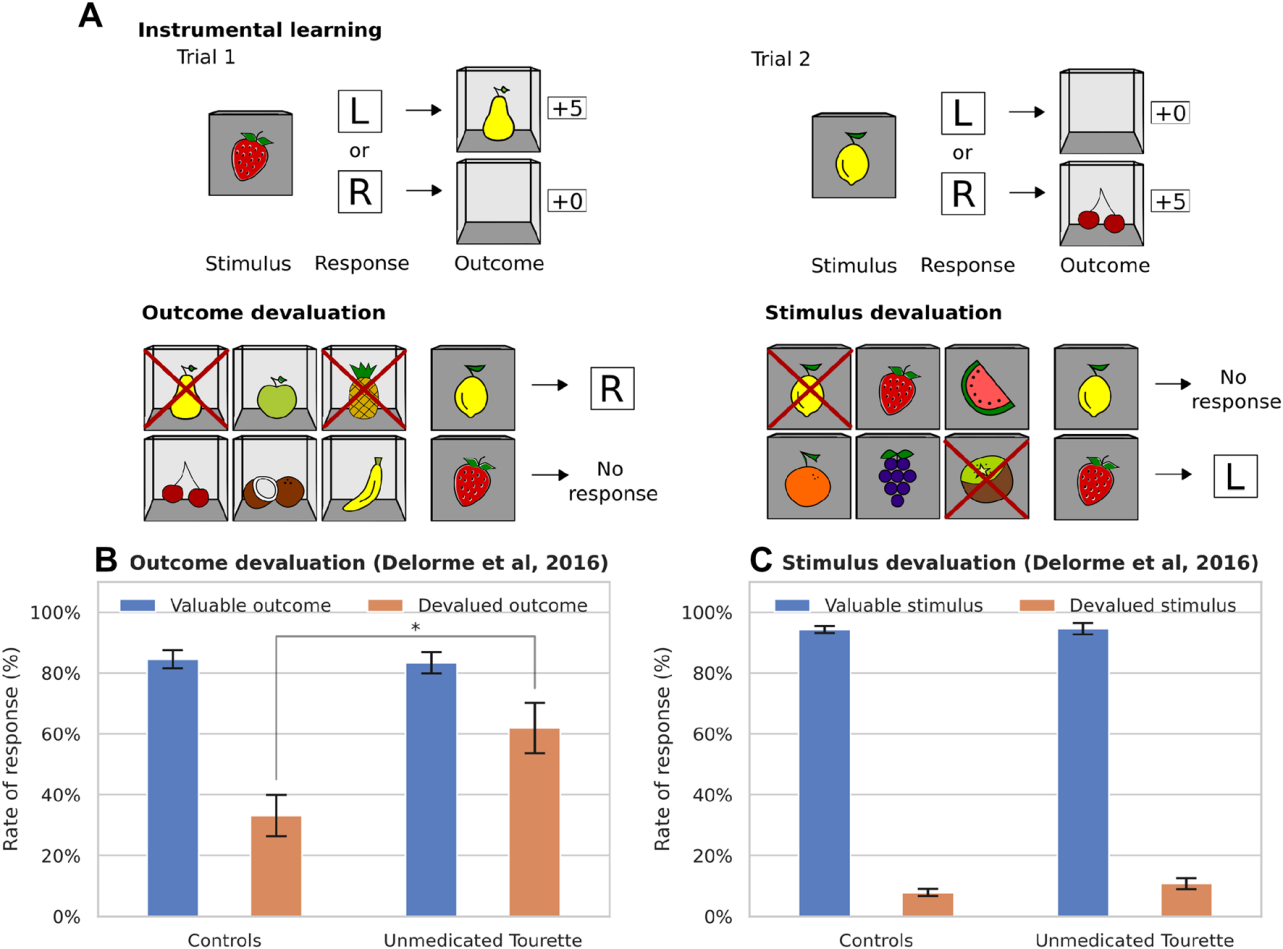
**A** Illustration of the task by Delorme et al. (2016). First, participants had to learn associations between 6 stimuli and 6 outcomes by pressing either a left (L) or right (R) button. There was a 100% contingency between stimuli, responses, and outcomes. After successful learning, 2 different outcomes were crossed out per block, marking them as devalued. Participants were instructed to no longer respond to stimuli associated with devalued outcomes. The devaluation of stimuli served as a response inhibition test. Participants were instructed to no longer respond to devalued stimuli. **B** and **C** Experimental results of unmedicated Tourette patients vs. healthy control subjects from the study by Delorme et al. (2016). While there was no difference in the responses towards devalued stimuli between groups (**C**), unmedicated Tourette patients responded towards stimuli associated with devalued outcomes at a significantly higher rate than healthy controls (**B**)

We tested our neuro-computational model with the task used by Delorme et al. (2016). In this task, participants had to learn stimulus-response-outcome associations (Fig. 1A). On each trial, a closed box labeled with a fruit icon was shown to the subject who was then asked to press the right or left button. A correct response was rewarded with points and an image of an open box that contained a fruit. If the subject pressed the wrong key, no points were awarded and an empty box was shown. Six different stimuli were linked to six different outcomes. After learning, Delorme et al. (2016) conducted a cognitive outcome devaluation and stimulus devaluation tests. In the outcome devaluation test, two of the possible outcome fruits were crossed out with an X, indicating that they would no longer award points. The participant was instructed to only press a key when a still-valued outcome could be obtained. The stimuli devaluation test followed the same procedure, however, two stimuli, instead of outcomes, were crossed out with an X at the beginning of each block.

Delorme et al. (2016) observed that both, the control group and the Tourette patients, could learn the task. However, during the outcome devaluation test, the patient group presented a higher rate of response towards devalued outcomes (Fig. 1B). Further, no significant difference between groups was found in the stimulus devaluation test (Fig. 1C). Thus, the difference in the outcome devaluation test could not be attributed to a general deficit in response inhibition of patients.

### Modeling framework

**Fig. 2:**
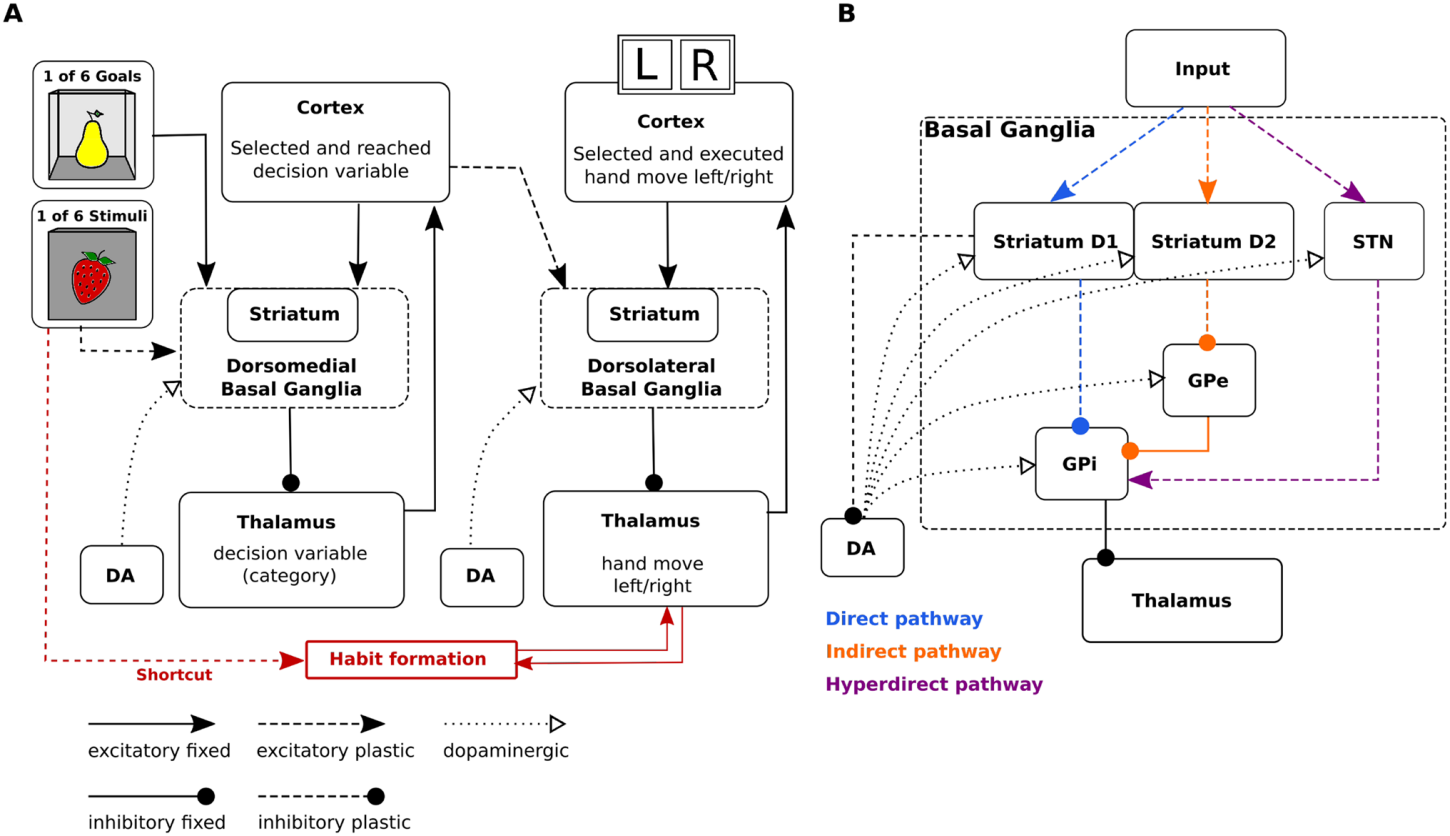
**A** Mapping of the task onto the model. In each trial the cognitive loop receives a stimulus signal and an associated goal signal as input, and outputs an objective sent to a premotor loop. A shortcut connects cortical neurons representing the input signal to thalamic cells of the premotor loop. The model response is read from the cortex. **B** Each of the two loops includes a direct, indirect, and hyperdirect pathway.* DA* dopamine,* GPi* internal globus pallidus,* GPe* external globus pallidus,* STN* subthalamic nucleus

We adapted the original hierarchical model of multiple cortico-basal ganglia loops by Baladron and Hamker (2020) to simulate the task of Delorme et al. (2016) (Fig. 2). The model is composed of a cognitive loop including the dorsomedial striatum and a motor loop including the dorsolateral striatum. Both loops are composed of populations of firing rate units and interact through overlapping cortico-striatal projections (Haber 2016).

The model proposes that an objective, such as obtaining points or food, is divided into a subset of decisions that finally leads to an expected outcome. These decisions are spread between the loops, each of them learning to select an intermediate objective at a different abstraction level, ranging from goals in the ventral striatum to motor commands in the putamen. Each loop provides an objective to the next hierarchical level, which in turn learns to determine the proper decisions to reach it. Further, the model includes cortico-thalamic shortcuts between loops, which are synaptic pathways that can bypass loops. Shortcuts are trained and monitored by the basal ganglia and are essential for habitual behaviors.

In the current implementation of the model, the dorsomedial loop receives a desired goal signal (here, a desired outcome fruit) and an associated stimulus signal (observed box with fruit icon) as inputs (Fig. 2A). Although the goal selection process is not explicitly modeled, we assume that it involves the limbic network (Groenewegen et al. 1997, 1999; Corbit et al. 2001; Balleine et al. 2003; Gönner et al. 2017), including the ventral striatum (Yael et al. 2019). The dorsomedial loop uses reward signals to learn to select a decision variable or cognitive category which is transferred to the dorsolateral loop as a reference signal. The dorsolateral loop then learns to select the appropriate hand movement. The model therefore distinguishes between the prediction of a state where the desired reward could be obtained and the required action to reach it. Such an organization provides multiple computational benefits such as transferring knowledge between tasks or simplifying the credit assignment problem (Baladron and Hamker 2020).

Cortico-thalamo-cortical pathways (Sherman and Guillery 2011), which in our model serve as a shortcut by bypassing the dorsomedial loop, directly link sensory information via cortical representations with hand movements (Fig. 2A). This shortcut is monitored and trained in a Hebbian manner by the basal ganglia through its output projections. In our previous work, we have shown how such a pathway can explain the emergence of habitual behavior and the ineffectiveness of outcome devaluation after overtraining (Baladron and Hamker 2020), as well as the effect of pallidotomy in Parkinsonian patients (Baladron and Hamker 2015; Schroll et al. 2014). The model has been implemented at a biologically plausible level using neural populations of rate coded neurons which follow a differential equation and thus allow to compute firing rate over time as specified in detail in the "Appendix".

## Results

### Role of cortico-thalamic shortcuts

**Fig. 3:**
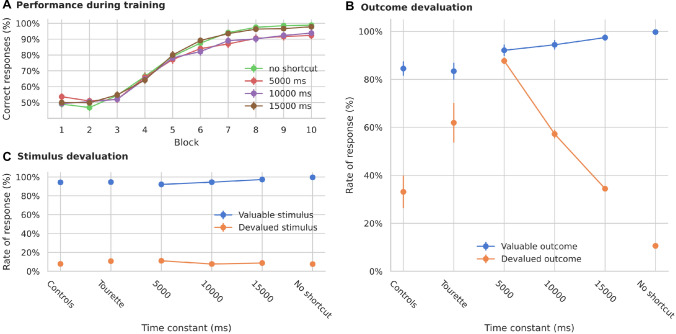
Simulated learning of the action-outcome associations of models with different time constants for learning the shortcut connections. **A** Learning performance over 10 blocks of 12 trials each. **B** Rate of response towards stimuli associated with devalued outcomes compared to still valuable outcomes. A smaller time constant produces more responses to devalued outcomes. **C** Rate of response towards devalued stimuli compared to still valuable stimuli. “Controls” and “Tourette” in panels **B** and **C** show the performance of human participants in the study by Delorme et al. (2016)

The shortcut connection links stimuli to the dorsolateral loop (Fig. 2A). In order to test the role of the shortcut, we ran experiments with different learning speeds in the shortcut connections, so that the weights changed by a different amount after each co-activation of the presynaptic and postsynaptic cells. One additional set of simulations was performed in which learning in the shortcut was fully disabled. As can be seen in Fig. 3A, all models initially select actions randomly, then gradually increase their performance, reaching a value higher than 90% after 8 blocks. This compares well with the results from Delorme et al. (2016), where both patients and controls reached a performance above 90% after the same number of trials.

Analogous to the experiments from Delorme et al. (2016), we then simulated outcome and stimulus devaluation. Outcome devaluation was simulated by canceling the goal signal reaching the dorsomedial loop on trials associated with a devalued outcome. This represents a lack of interest in the possible reward. Stimulus devaluation was simulated by reducing the input stimulus signal on trials associated with a devalued stimulus. This represents the fact that the stimulus is still observed, but not attended. Two of all possible outcomes or stimuli were devalued in each of the 6 blocks.

Although all versions of the models show similar performance during training (Fig. 3A), outcome devaluation has different effects. Models with faster learning in the shortcut (smaller time constant, $\tau _w$ in Eq. 13) select devalued outcomes more frequently than models with medium or slow learning speeds (Fig. 3B). The results on the stimulus devaluation test however show no significant difference between the models (Fig. 3C, on a permutation test with all combinations, the smallest *p* was 0.06 between tau 12,000 and 6000 and the largest was 0.95 between tau 14000 and 6000).

The difference between models with different learning speeds compares well to observed differences by Delorme et al. (2016) between Tourette patients and controls. On outcome devalued trials, patients had a significantly higher rate of response towards devalued outcomes, suggesting that patients relied more on habitual behavior than control subjects. Further, they reported no significant difference between groups in the stimulus devaluation test.

Our results suggest that faster learning in the cortico-thalamic shortcut can explain the difference between controls and patients in the task of Delorme et al. (2016). As in our previous simulations (Baladron and Hamker 2020), the development of habitual behavior relies on these connections, which bypass the goal analysis done by the dorsomedial loop. To further test this hypothesis, we ran an additional set of simulations in which learning in the shortcut was completely disabled. Models without plasticity in the cortico-thalamic shortcut are unable to develop habits and therefore have a much lower response rate to devalued outcomes (Fig. 3B). However, their performance on learning the task is similar to that of models with enabled shortcut learning. This confirms the habit learning framework of Baladron and Hamker (2020): habits emerge by learning shortcuts.

In summary, an increase in the shortcut learning speed can drive the model to rely more on habitual behavior, providing a possible explanation to the enhanced habit formation in Tourette patients. Such a change in the model could however emerge from different pathological causes, such as a direct modification of the shortcut connections or from a more indirect aberrant modulation of the components of the learning rule. In the following, we explore whether such aberrant learning may arise from hypothesized pathophysiologies of Tourette syndrome.

### Enhanced dopaminergic modulation

It has been suggested that tics in Tourette syndrome are caused by dopaminergic dysfunction. While the exact anomaly is still debated, theories tend to link higher concentrations of dopamine in the axon terminals to the symptoms (Buse et al. 2013). Following these results, increased habitual responses may be produced due to a strengthened direct pathway and not necessarily through abnormal shortcuts as we propose here. However, in the context of our model, altered dopamine signaling can indirectly affect the shortcut’s behavior by either changing the output of the basal ganglia that trains the shortcut, or by modulating the shortcut’s feedback to the striatum, creating a bias towards the action selected by the shortcut.

Dopamine is known to have two different effects on striatal cells (Gerfen and Surmeier 2011). First, it modulates the activation of cells depending on the dopamine receptor being stimulated (Surmeier et al. 2007). D1 receptor signaling increases the activation while D2 receptor signaling decreases it. Second, dopamine regulates plasticity (Wickens 2009). An increase in the dopamine level enhances long-term potentiation in cells expressing D1 receptors and long-term depression in cells expressing D2 receptors (Shen et al. 2008; Fisher et al. 2017). We investigate both mechanisms separately in our model to better understand the effect of each on the habitual responses of Tourette patients.

#### Effects of altered response modulation

**Fig. 4:**
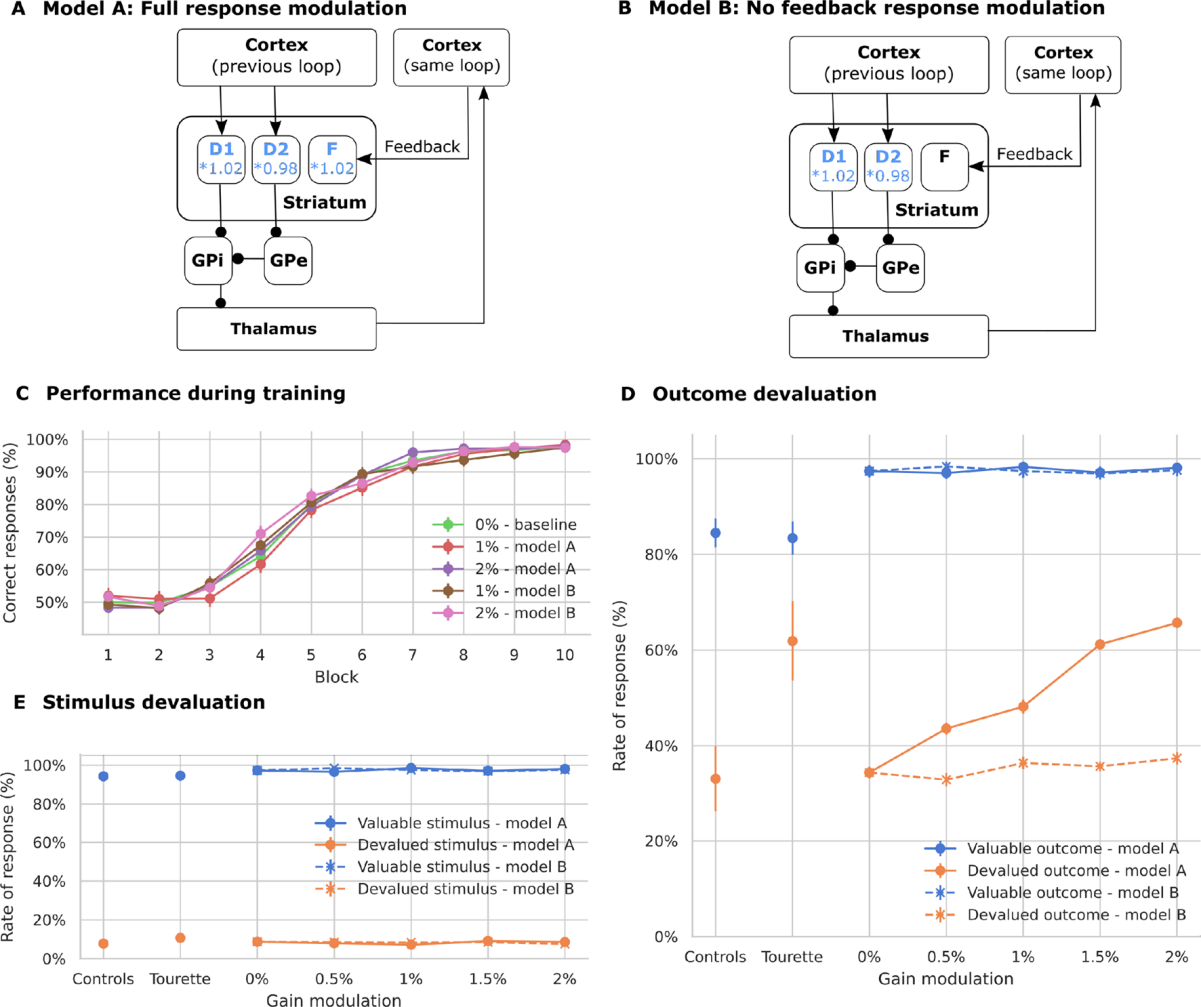
Simulated learning of action-outcome associations of models with dopamine-mediated response modulation of striatal firing rates. All other connections, including the pathway via the subthalamic nucleus (STN) are modeled as before. **A** To test the dopamine-dependent rate modulation hypothesis, the output rate of striatal cells is multiplied by a fixed factor (model A). **B** In model B, the response modulation is not applied on the feedback connections. **C** Learning performance over 10 blocks of 12 trials each. All models show a similar performance. **D** Responses towards stimuli associated with still valuable and devalued outcomes. Only models with full response modulation, including the feedback pathway, show an increased rate of responses. **E** Responses towards valuable and devalued stimuli. “Controls” and “Tourette” in panels** D** and** E** show the performance of human participants in the study by Delorme et al. (2016)

We first modeled the effect of dopamine on the firing rate of striatal cells. Increased dopamine levels were implemented by introducing a scaling factor for the membrane potential to the firing rate ($S_f$ in Eq. 1, Fig. 4A). With higher levels of tonic dopamine, the excitability of cells in the direct pathway is increased, while it is decreased in the indirect pathway. The parameters of the plasticity rule were unaffected, and the time constant was set to 15,000 ms (Fig. 3B).

There are two possible ways in which dopamine-based response modulation can affect habitual responses in the model. The first option is that the imbalance of the indirect and direct pathways disrupts the outputs of the basal ganglia to the thalamus, which are used to train the shortcut. The second option is that the feedback to the striatum is increased, thereby biasing the response. In order to disentangle the effects, we performed an additional experiment in which we removed the response modulation on those cells receiving cortical feedback (Fig. 4B). These feedback projections transmit shortcut activation to the dorsolateral loop and enhance it through the direct pathway. In this condition, response modulation is only present for those cells that receive exclusively inputs from the previous loop through overlapping cortico-striatal projections.

Both options of the model with dopamine-dependent response modulation as well as the control model learn the task and reach similar performance levels (Fig. 4C). The models however vary in the rate of responses to devalued outcomes (Fig. 4D): A dopamine-dependent modulation of the firing rate leads to more responses to devalued outcomes (significant difference between modulation level 1.02 and control, permutation test *p* < 0.001), similar to those of unmedicated Tourette patients as reported by Delorme et al. (2016). However, this effect depends on the dopamine-dependent modulation of the feedback signal, as models without such a modulation do not increase their responses to devalued outcomes (Fig. 4D, no significant difference, *p* = 0.11), while not affecting the learning of the task (Fig. 4C).

Shortcut connections can therefore make the model rely more on habitual behavior, not only when the speed of plasticity in shortcuts is increased, but also when the impact of the shortcut on the basal ganglia circuits is increased through a dopamine-dependent modulation of striatal activity.

#### Effects of altered plasticity modulation

**Fig. 5:**
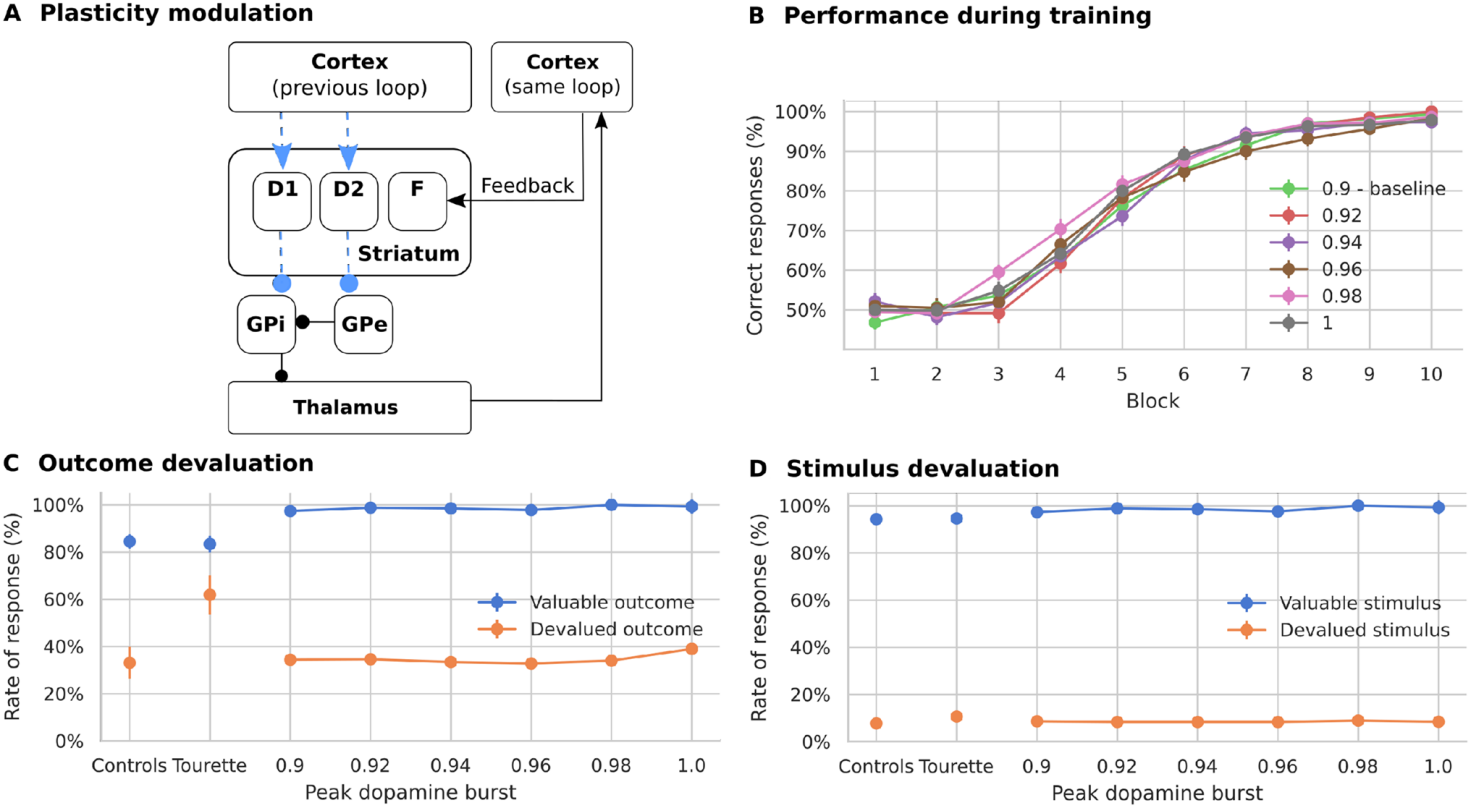
Simulated learning of action-outcome associations of models with dopamine-mediated up- and down-regulation of plasticity of cortico-striatal synapses. **A** The effect of the dopamine signal on plasticity is enhanced. Connections affected by the change are shown in dashed blue lines. In each model, the size of the phasic increase in the dopamine signal after receiving reward is different. The baseline dopamine used to compute the level of a phasic change is lowered in order to increase the maximum amplitude reached by the phasic response (see Eq. 2 and Sect. 5.3). **B** Learning performance over 10 blocks of 12 trials each. All models present similar performance. **C** Responses towards stimuli associated with still valuable and devalued outcomes. Increasing the amplitude of the dopamine bursts does not significantly change the learning behavior. **D** Responses towards valuable and devalued stimuli. “Controls” and “Tourette” in panels **C** and **D** show the performance of human participants in the study by Delorme et al. (2016)

Second, we tested whether dopamine modulation of cortico-striatal plasticity could also indirectly affect the shortcut and increase habitual responses. Thus, we increased the impact of dopamine in the learning period after reward delivery, thereby amplifying long-term potentiation in D1 cells and long-term depression in D2 cells (Fig. 5A).

Models with increased plasticity modulation also learn the task (Fig. 5B), but their response rate towards devalued outcomes is similar to control models (Fig. 5C, no significant difference, permutation test between controls and models with dopamine peak 1.0 with $p>0.01$). Further, all versions show a small and similar rate of responses to devalued stimuli (Fig. 5D, in a permutation test for all combinations the minimum *p* was 0.13 between peak dopamine burst 0.94 and 0.92, and the maximum *p* was 0.95 between peak dopamine burst 0.98 and 0.94).

Thus, our model of increased dopamine-mediated up- and down-regulation of plasticity does not lead to pronounced responses towards devalued outcomes.

### Reduced local inhibition in the striatum

The second major hypothesis regarding Tourette pathophysiology involves reduced striatal inhibition (Bronfeld and Bar-Gad 2013; Vinner et al. 2017). We therefore tested whether reducing the weights between striatal inhibitory projection neurons would produce any change in the rate of responses to devalued outcomes and could account for the observations of Delorme et al. (2016).

#### Effects of reduced inhibition in the dorsomedial striatum

**Fig. 6:**
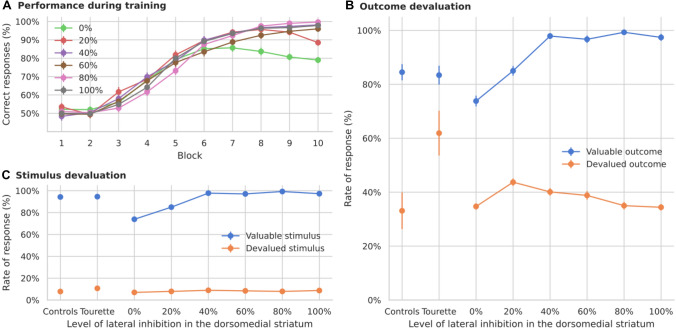
Simulated learning of action-outcome associations of models with reduced local striatal inhibition in the dorsomedial loop. **A** Learning performance over 10 blocks of 12 trials each. In each model the weight is reduced by a fraction of their original value (from no inhibition, 0%, to control, 100%). When the inhibition is completely removed, the model becomes unstable. **B** Responses towards stimuli associated with valuable and devalued outcomes. The rate of response to devalued outcomes shows a small increase when inhibition is strongly reduced. **C** Responses towards valuable and devalued stimuli. Models with a strong reduction show less frequent responses to valuable stimuli. “Controls” and “Tourette” in panels **B** and **C** show the performance of human participants in the study by Delorme et al. (2016)

The performance of the model with lowered inhibitory connections in the dorsomedial striatum is robust to a decrease in the weights down to 40% of the original level (Fig. 6A). Models with no local inhibition become unstable at block 5 during learning and do not learn the task well. Models with reduced weights show a trend towards an increased rate of responses towards devalued outcomes (Fig. 6B, significant difference, *p* < 0.001). However, the response rate is lower than for patients tested by Delorme et al. (2016). The rate of response to devalued stimuli is similar in all cases (Fig. 6C, permutation test with all combinations, the maximum *p* was 0.82 between 40% inhibition and 80% inhibition, the minimum *p* was 0.15 between 20% inhibition and 40% inhibition).

Models with a strong reduction of dorsomedial striatal inhibition show a higher variability in the weight matrix learned by the shortcut during the task. Although the mean weight value in both the control models (100% inhibition) and those with weights decreased to 40% is the same (0.65), a significant difference occurs in their standard deviation (difference of 0.001, *p* = 0.004). This indicates that models with reduced inhibition produce a variability in shortcut strength that can make them more dependent on habitual behavior. Increased shortcut variability changes the balance in the baseline of the thalamus, allowing the basal ganglia to take over the control through its inhibitory projections. Therefore, unlike the effect of firing rate modulation, reduced inhibition can affect habitual responding by modulating shortcut plasticity directly, and not via the feedback connection. Its overall impact however is much smaller.

#### Effects of reduced inhibition in the dorsolateral striatum

**Fig. 7:**
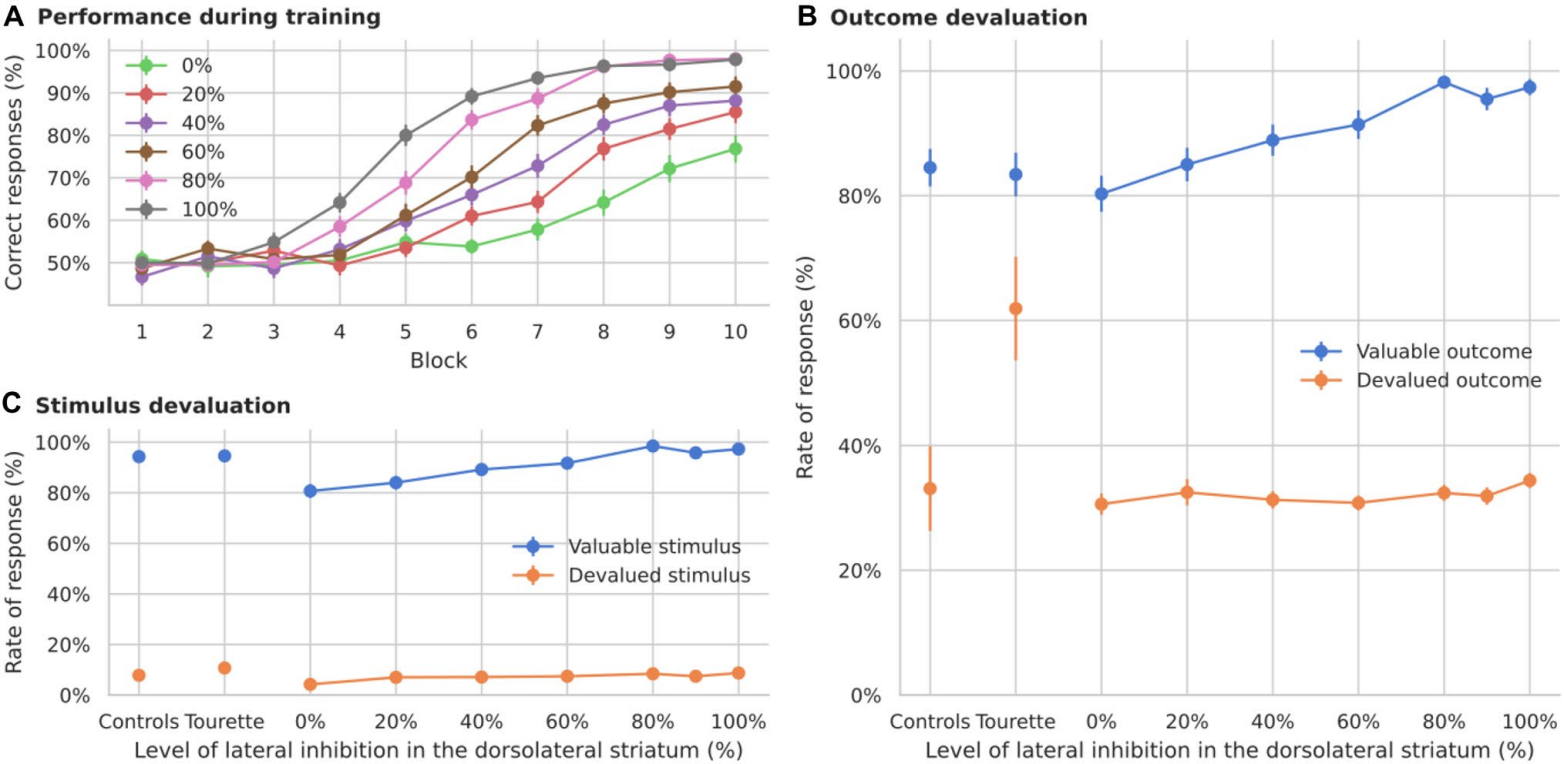
Simulated learning of action-outcome associations of models with reduced local striatal inhibition in the dorsolateral loop. **A** Learning performance over 10 blocks of 12 trials each. Only models with a small reduction in inhibition show a performance similar to controls. **B** Responses towards stimuli associated with valuable and devalued outcomes. Different levels of local striatal inhibition in the dorsolateral loop do not affect the rate of response to devalued outcomes. **C** Responses towards valuable and devalued stimuli. Models with a strong reduction show less frequent responses to both, valuable and devalued stimuli. “Controls” and “Tourette” in panels **B** and **C** show the performance of human participants in the study by Delorme et al. (2016)

We also reduced the inhibition in the striatum of the dorsolateral loop following the same procedure as for the previous loop. This reduction has a stronger effect on the learning performance of the model (Fig. 7A) and only those models with a slight reduction of inhibition reach a performance similar to control models and human subjects. Reduced inhibition in the dorsolateral striatum, however, does not lead to an increase in response rate to devalued outcomes comparable to Tourette patients in Delorme et al. (2016) (Fig. 7B).

Consequently, our model predicts only a modest increase in habitual behavior with reductions of lateral inhibition in the dorsomedial striatum, but no effect to altered inhibition levels in the dorsolateral striatum.

## Discussion

The neurobiological underpinnings of Tourette syndrome are still not completely clear. Two main lines of research focus on explaining tic generation through either imbalanced inhibition (Kalanithi et al. 2005; Kataoka et al. 2010; Vinner et al. 2017) or anomalies of dopamine transmission (Maia and Conceição 2017, 2018). Based on the suggested commonalities of tics and habits, we propose a neurocognitive model of enhanced habit formation in Tourette syndrome. The model’s difference in behavior under pathological and default parameter configurations offers new avenues to understand Tourette pathophysiology and complements traditional views.

In our model, two hierarchically organized cortico-basal-ganglia-thalamo cortical loops simulate the increased engagement of habitual behavior by Tourette patients. Sensory inputs can either drive neurons in the dorsomedial striatum directly or reach the dorsolateral striatum via a cortico-thalamic shortcut. We here propose that enhanced habit formation in Tourette patients, as observed by Delorme et al. (2016), may be grounded in aberrant activation within cortico-thalamic shortcut connections. Models with faster learning in the shortcut produce similar data as Tourette patients. To better link aberrant learning with the potential pathophysiology of Tourette, we investigated two hypotheses – enhanced dopamine signaling and striatal disinhibition. Enhanced dopamine signaling modulates the activation of the shortcut through the closed loop formed by the direct pathway and feedback cortical connections. Reduced striatal inhibition introduces a high variability in the shortcut. Both changes increase the amount of habitual responses, mimicking the behavior of Tourette patients reported by Delorme et al. (2016).

### Relation between Tic formation and habit formation

In this work, we primarily address enhanced habit formation of Tourette patients in a cognitive task, and not necessarily tic generation. Yet as our model simulations have shown, altered shortcut behavior can be indirectly produced through the two anomalies that have been discussed in the context of tic formation as well.

Tics have been compared to habits from both a cognitive-behavioral and neuroscientific perspective (Leckman and Riddle 2000; Maia and Conceição 2017; Delorme et al. 2016; Shephard et al. 2019; Beste and Münchau 2018). Our model appears consistent with the cognitive framework of altered perception-action binding in Tourette patients (Beste and Münchau 2018), as the shortcut binds perceptual states to motor actions via the basal ganglia. Kleimaker et al. (2020) demonstrated that Tourette patients show an increased perception-action binding. This may be regarded as a surplus of actions (Beste and Münchau 2018), some of which become habits due to an increased propensity towards habit learning (Delorme et al. 2016) and reward learning (Palminteri et al. 2009, 2011). Specifically, tics may be habitual responses towards unpleasant somatosensory internal stimuli, the so-called premonitory urges. The termination of the urge through tic execution can be viewed as avoiding punishment, making future tic execution more likely (Brandt et al. 2016; Capriotti et al. 2014; Kwak et al. 2003), and after repeated execution automatic and habitual. Yet it should be noted that the typical sequence of symptom onset is conflicting with this view. Children typically first report about urges around three years after tic onset (Openneer et al. 2019). This could however also be attributed to missing awareness for urges and unreliable reporting. Finally, habit reversal training is a promising therapy option that views tics as maladaptive habits (Dutta and Cavanna 2013): it tries to identify the preceding urge (= stimulus) and replace the tic with an alternative action instead of suppressing it.

The learning and execution of tics and habits in Tourette syndrome may be accelerated due to increased phasic dopamine bursts and dips onto cortico-striatal projections (Maia and Conceição 2017; Conceição et al. 2017). At the same time, patients may have an increased propensity to execute such learned tics due to increased activation of the Go (direct) relative to No-Go (indirect) pathway, as higher tonic dopamine levels increase and decrease the gain of D1 and D2 medium spiny neurons, respectively (Maia and Conceição 2017; Conceição et al. 2017). However, according to this framework, both tic learning and tic execution take place in the dorsolateral motor loop through the putamen, without the involvement of multiple loops or shortcuts. This view assumes the traditional perspective of parallel loops that localizes habitual actions in the sensorimotor dorsolateral loop (Yin and Knowlton 2006). This organization however has been challenged by models which consider recent experiments regarding cortico-striatal projections (Baladron and Hamker 2020; Balleine et al. 2015; Collins and Frank 2013). A unique feature of our approach is the hierarchical organization of the multiple cortico-basal ganglia-thalamo-cortical loops.

Another line of evidence that has to be taken into account when modeling habitual behavior, comes from animal models: habit learning does not only involve the basal ganglia, but also critically depends on cortical areas. For instance, rats with lesions of the infralimbic cortex are unable to develop habits (Killcross and Coutureau 2003). Further, the execution of established habits can be prevented if the same area is inactivated after learning (Smith et al. 2012; Coutureau and Killcross 2003). Thus, the medial prefrontal cortex may be central to both learning and execution of habits. Our model explicitly includes a cortico-thalamo-cortical shortcut to model this dependence and suggests that enhanced habit formation in Tourette syndrome may be explained by increased speed of learning in this shortcut. Given the analogy of tics and habits, we encourage future models to include such shortcuts. Their dysfunction could be investigated as a potential pathophysiological feature that contributes to the learning and execution of tics, and not just habits, in Tourette syndrome.

Although apparently weak, there is evidence of connections from the infralimbic cortex to the ventromedial area of the thalamus which is known to be involved in motor control (Hurley et al. 1991). Further, the pre-frontal cortex has been reported to form reciprocal connections not only with the mediodorsal thalamus but also with the ventral motor thalamic nuclei (Sieveritz et al. 2019). Collins et al. (2018) found through optogenetic stimulation that the prefrontal cortex can strongly drive both the mediodorsal and ventromedial thalamus. In our model, direct connections from the infralimbic cortex to the initial loops of the hierarchy (limbic system, nucleus accumbens) could help in reducing the processing within the loops once shortcuts are learned. This however, needs to be studied in future modeling experiments. It should be noted that the infralimbic cortex has been anatomically linked mainly with areas of the limbic system, such as the nucleus accumbens, amygdala or hypothalamus (Vertes 2004; Barker et al. 2014). Its corresponding area in humans is still under discussion, with Brodmann areas 25 and 32 in the ventromedial prefrontal cortex as prominent candidates (Roberts and Clarke 2019). Therefore the exact location of the suggested shortcuts in the human brain is still uncertain.

Around half of Tourette patients also present obsessive compulsive disorder (Goodman et al. 2006). Young patients with comorbid Tourette syndrom and obsessive-compulsive behavior have more severe tics (Lebowitz et al. 2012) and rely more on habitual behavior (Gillan et al. 2014). Further, current studies relate obsessive-compulsive disorder with a disruption in the balance between goal-directed behavior and habits (Gillan et al. 2011). According to our model, all these symptoms could be associated to shortcut malfunctions. A tentative compromise may be implemented by a two-step model of Tourette, where tics initially emerge by reduced levels of inhibition onto striatal projection neurons and then become manifested by enhanced habit formation.

### Role of dopamine

The role of dopamine further supports the putative link between habits and tics. On the one hand, dopaminergic disturbances present a central suspected pathophysiological feature of Tourette syndrome (Buse et al. 2013; Maia and Conceição 2017, 2018). Mice with excessive striatal dopamine show frequent rigid and complex action patterns and serve as an animal model of the Tourette syndrome (Berridge et al. 2005). On the other hand, dopamine takes a critical role during learning of habitual behaviors, although its influence on the execution of learned habits may diminish with growing cortical control (Ashby et al. 2010).

Our results suggest that excessive dopamine may increase habitual responses through an enhancement of the shortcut’s feedback to the striatum. Accordingly, it has recently been shown that rats develop habitual responses faster when they were exposed to the dopamine precursor levodopa (Gibson et al. 2020). Accelerated habit formation has also been observed in rats whose dopamine levels were increased through amphetamine sensitization (Nelson and Killcross 2006). The inability of animals to form habits following lesions of the nigrostriatal dopamine system (Faure et al. 2005), the dorsolateral striatum (Yin et al. 2004), or the infralimbic cortex (Killcross and Coutureau 2003; Coutureau and Killcross 2003) reveals critical brain regions for habit formation and its dependence on dopamine. Experiments on rats further show that behavior becomes less dependent on dopamine with extended training (Choi et al. 2005), which could correspond to control being transferred from the loops to the shortcut. Indeed, it has been hypothesized that dopamine only affects the early learning of habits (Ashby et al. 2007). Assuming a link between tics and habits, dopamine-modulating medication may thus be more effective in preventing the learning of new tics instead of suppressing existing tics.

### Tourette treatments may affect shortcut connections

In our model with multiple loops, habitual behavior does not emerge if plasticity in the shortcut projections is disabled. Habits are not released from the dorsolateral loop alone, because thalamic cells are biased by a fast transmission of visual inputs via cortico-thalamic shortcut projections. The association between the respective cortical and thalamic cells is slowly acquired over repeated trials, with the basal ganglia providing a teaching signal for the shortcut. Our simulation results suggest that this slow incremental learning process may be accelerated in the case of Tourette syndrome, benefiting the fast development and consolidation of habits which can manifest as tics. The effectiveness of habit reversal training (Dutta and Cavanna 2013) and comprehensive behavioral intervention (Petruo et al. 2020) in treating tics may be explained by a rewiring in these shortcut projections. The initially learned maladaptive behavior (tic) can be replaced by another action if the connection pattern between cortical and thalamic cells in the shortcut can be modified.

A common target for deep brain stimulation in Tourette patients is the thalamic centromedian-parafascicular (CM-Pf) region (Schrock et al. 2015; Britoa et al. 2019; Xu et al. 2020). According to our approach, the thalamus is a critical element of the shortcut, and indeed Tourette patients had increased basal ganglia-cortical and thalamo-cortical connectivity in a recent fMRI study (Ramkiran et al. 2019). Stimulation of the thalamus could therefore interfere with the spread of information through the cortico-thalamo-cortical pathway.

### Evidence for shortcuts in the brain

Our modeling results suggest that shortcut connections are crucial to learn and engage in habitual behavior. However, as this prediction is novel, there are no systematic studies that focused on the identification of shortcuts. Nevertheless, several studies provide consistent evidence for our hypothesis, which we summarize here.

The proposed shortcut structure requires that the involved cortical areas project not only to the thalamic part from which they receive an afferent projection, but also to other thalamic parts. McFarland and Haber (2002) already emphasized that the thalamus not only has reciprocal connections with cortex, but also non-reciprocal, so called feedforward connections, to relay information between different cortical sites. Corticothalamic projections are more extensive than their thalamocortical counterpart, with each thalamic nucleus combining a reciprocal and non-reciprocal component (Haber and Calzavara 2009). For example, corticothalamic neurons in the lower part of layer 6 of the rat’s barrel cortex project non-reciprocally to several barreloids within the ventral posteromedial nuclei of the thalamus (Deschenes et al. 1998). Medial prefrontal cortex projects to both the central mediodorsal and the ventral anterior nuclei of the thalamus, and the pars oralis of the ventro lateral nucleus has a non-reciprocal afferents from rostral motor regions (McFarland and Haber 2002; Haber and Calzavara 2009). Similarly, using a combination of optogenetics and tracing experiments, Collins et al. (2018) revealed that pre-frontal cortex project to both the mediodorsal and ventromedial thalamus.

From a more thalamic-centric view, anatomical patterns linking a single thalamic part to multiple cortical areas also support our framework. Haber and Calzavara (2009) reported that the central mediodorsal nucleus of the thalamus is reciprocally connected to the lateral and orbital prefrontal cortex and in addition receives input from the medial prefrontal cortex. Similarly, the ventral anterior nucleus is reciprocally connected to both the dorsal premotor cortex and the caudal dorsolateral prefrontal cortex, while additionally it is linked to medial prefrontal areas. Such patterns are also present in more motor related parts: the ventral lateral nucleus is reciprocally connected with caudal motor areas, but it is also non-reciprocally connected with rostral motor cortical regions.

The idea of cortico-thalamo-cortical pathways supporting intra-cortical communication is not new (Sherman and Guillery 2011; Haber and Calzavara 2009; Zajzon and Morales-Gregorio 2019). Cortico-cortical communication can be canceled or reduced by lesioning the thalamus (Theyel et al. 2010; Soares et al. 2004), but its function is not well understood. We here propose how this interaction could be controlled by the basal ganglia and the effects it may have on the development of habitual behavior. The functional relation between cortex and thalamus may be even more complex as there is evidence for multiple subtypes of cortico-thalamic and thalamo-cortical synapses depending on the layer of origin and axonal properties of the presynaptic cell (Rockland 2015; Sherman and Guillery 2011). Based on our modeling results, we recommend that these pathways should be identified and studied further in the context of habitual behavior.

### Limitations and open issues

Although our model could recreate the task behavior of patients observed by Delorme et al. (2016), we did not include any explicit representation of tics, the core symptom of the Tourette syndrome. We already discussed the close resemblance of habits and tics (Leckman and Riddle 2000). Our model however could be complemented with a direct representation of tics in order to simulate a wider range of experiments. For instance, Caligiore et al. (2017) proposed a neuro-computational model of a single loop in order to explain tic generation in a pharmacological monkey model of motor tics studied by McCairn et al. (2013). According to their model, tics are generated by a dysfunctional interaction between the cortico-basal-ganglia loops and the cerebellum. In their simulations, enhanced phasic bursts of dopamine made the basal ganglia overly sensitive to cortical noise, producing undesired activation of the premotor cortex which is understood as tic initiation. Although their model uses similar firing rate units and has a comparable structure to ours, it does not include plasticity and can therefore not learn to solve any task.

A central finding in support of the hypothesis of reduced striatal inhibition is the loss of interneurons observed in stereological analyses of post-mortem brains of Tourette patients (Kalanithi et al. 2005; Kataoka et al. 2010). As our model of the striatum is composed only of medium spiny neurons, we have approximated the loss of inhibitory interneurons by a reduction of local inhibitory connections. However, the loss of inhibitory interneurons may result in a more complex change than approximated in the present model version.

Delorme et al. (2016) not only reported the behavioral results replicated here, but also used diffusion tensor imaging to study the structural connectivity within the basal ganglia. They found that a higher amount of responses to devalued outcomes was correlated with an increase in the connectivity of the motor network. Possible effects of shortcuts however were excluded from their analysis as only the posterior sensorimotor putamen and the anterior caudate nucleus were used as seeds. Based on our results we suggest to further include cortical or thalamic areas as seed regions in future experiments.

A recent idea regarding the pathophysiology of Tourette suggests that abnormalities are not limited to the basal ganglia circuits, but extend to the social behavior network including the medial amygdala-bed nucleus, the hypothalamic medial preoptic area, the anterior hypothalamus, the ventromedial hypothalamus, the lateral septum, and the midbrain periaqueductal grey-central grey (Albin 2018). According to this hypothesis, tics result from altered interactions between this network and the dopaminergic cells projecting to the ventral striatum. However, our current implementation neither includes the social behavior nuclei nor the ventral basal ganglia loop and therefore can currently not be used to test this hypothesis.

As a final remark, our model has been originally developed to explain habit formation in animals (Baladron and Hamker 2020). Habits such as those investigated by Delorme et al. (2016) however, refer rather to a more cognitive outcome-insensitive behavioral control. Humans appear in general more sensitive to outcome-devaluation and thus, less sensitive to habits (de Wit et al. 2018). Despite these discrepancies between human and animal studies of habit formation, our model may rather help to understand such differences, as habits imposed by the shortcut could be diminished by cognitive control.

## Data Availability

The datasets generated during and/or analyzed during the current study are available from the corresponding author on request.

## Appendix

### Model details

The cortex - basal ganglia model is based on the one proposed by Baladron and Hamker (2020), which further extends that of Schroll et al. (2014) to include multiple loops. Each basal ganglia loop includes the direct pathway through the striatum and the internal globus pallidus (GPi), the short indirect pathway through the striatum and the external globus pallidus (GPe), and the hyperdirect pathway through the STN (Fig. 2B). Neuronanatomical evidence for the pathway structure of the original model has been reviewed in Schroll and Hamker (2013).

Neural populations were modeled by a number of rate coded neurons which follow the differential equation:

1 $$\begin{aligned} \begin{aligned} \tau \frac{dm_j}{dt} + m_j&= \sum _{i \in N_e} w_{ij}r_i - \sum _{i \in N_i}w_{ij}r_i + B + \epsilon _j,\\ r_j&= (m_j)^+S_f. \end{aligned} \end{aligned}$$

where $m_j$ is the membrane potential of cell *j*, $r_j$ the firing rate of cell *j*, $\tau$ a time constant, $w_{ij}$ the weight between presynaptic cell *i* and postsynaptic cell *j*, $N_e$ are the cells with an excitatory synapse to cell *j*, $N_i$ are the cells with an inhibitory synapse to cell *j*, *B* is a baseline activity and $\epsilon _j$ is a noise term drawn from a uniform distribution, $()^+$ converts negative numbers to 0. $S_f$ is a scaling factor used to simulate disturbed altered response modulation. On control models it is set to 1.

The striatum of each loop is divided into two main groups: One for D1 dopamine receptor expressing cells, and one for D2 dopamine receptor cells. Each loop contains an additional smaller set of cells that implement cortical feedback (Fig. 2A). These neurons have afferent connections only from the cortical cells of their corresponding loop and project to both the GPi and the GPe. A similar mechanism was used in the original model (Schroll et al. 2014) to implement thalamic feedback and facilitate learning in each pathway.

Learning in the cortico-striatal projections follows a dopamine-modulated covariance learning rule:

2 $$\begin{aligned} \tau _w \frac{dw_{ij}}{dt}=f_{DA}(DA(t)-B_{DA})C_{ij}-\alpha _j(r_{j}-{\overline{r}}_\text {POST})^2 \end{aligned}$$

where $w_{ij}$ is the weight of the synapse between presynaptic cell *i* and postsynaptic cell *j*, $f_{DA}$ implements dopamine modulation and it is a function of the difference between the current dopamine level *DA*(*t*) and the baseline dopamine level $B_{DA}$, $C_{ij}$ is a correlation measure and $\alpha _j(r_{j}-{\overline{r}}_\text {POST})^2$ a normalization term that limits the weight growth.

The function $f_{DA}$ ensures that a phasic dopamine increase enhances long-term potentiation in D1 receptor expressing cells and long-term depression in D2 receptor expressing cells (Shen et al. 2008; Villagrasa et al. 2018). The expression for this function is:

3 $$\begin{aligned} \begin{aligned} f_{DA}(x)&= {\left\{ \begin{array}{ll} (T_{d}\cdot Kb\cdot x) &{} if\ (T_{d}\cdot x)>0\\ (T_{d}\cdot Kd\cdot x) &{} if\ (T_{d}\cdot x)<0 \wedge T_d\cdot C>0\\ 0 &{} else \end{array}\right. }, \end{aligned} \end{aligned}$$

where $T_d$ controls the effect of dopamine, *Kb* and *Kd* the size of increases and decreases of the weights. $T_d$ is 1 on projections to D1 expressing cells and -1 to D2 expressing cells.

The correlation measure $C_{ij}$ is defined such that only connections between active cells are subject to plasticity. In the dorsomedial loop, it follows:

4 $$\begin{aligned} C_{ij} = (r_i-{\overline{r}}_\text {PRE}-\gamma _\text {PRE})(r_j-{\overline{r}}_\text {POST} - \gamma _\text {POST})^+ \end{aligned}$$

where $r_i$ and $r_j$ are the rates of the presynaptic and postsynaptic cells, ${\overline{r}}_\text {PRE}$ and ${\overline{r}}_\text {POST}$ are the mean firing rates of the presynaptic and postsynaptic populations and $\gamma _\text {PRE}$ and $\gamma _\text {POST}$ are thresholds.

The correlation measure is different in the dorsolateral striatum, where a trace ($Tr_j$) is required. The trace allows plasticity to occur only between cells that were active during the period in which an action was selected. Active cells during this period may be different to those which become active once dopamine increases or decreases due to the integration of environmental information done by cortical cells. Correlation is then defined as:

5 $$\begin{aligned} \begin{aligned} C_{ij}&= (r_i-{\overline{r}}_{PRE}-\gamma _\text {PRE})(Tr_j-{\overline{Tr}}_\text {POST} - \gamma _\text {POST})^+ \\ \tau _T\frac{dTr_j}{dt}&= r_j - Tr_j \end{aligned} \end{aligned}$$

The normalization term in equation 2 is only active if the activity of the postsynaptic cell is larger than a fixed threshold ($m^\text {MAX}$). This is controlled through $\alpha$ which is governed by the following differential equation:

6 $$\begin{aligned} \tau _{\alpha }\frac{d\alpha _j}{dt} + \alpha _j = (r_j - m^\text {MAX})^+ \end{aligned}$$

The model also includes plasticity in the striato-pallidal connections. Different than cortico-striatal connections, the learning rule in these projections ensures that plasticity occurs only when the activity of the pallidal cell is below the mean. This is necessary because for the selection of an objection/action a decrease of the firing rate is required as opposed to an increase. The weight change then follows:

7 $$\begin{aligned} \tau _w\frac{dw_{ij}}{dt} = f_{DA}(DA(t) - B_{DA})\cdot C_{ij} - \alpha _j(C_{ij})^+ \end{aligned}$$

with $f_{DA}(x)$ and $\alpha _j$ defined in equation 3 and with:

8 $$\begin{aligned} C_{ij} = (r_i-{\overline{r}}_\text {PRE}-\gamma _\text {PRE})^+({\overline{r}}_\text {POST}-r_j-\gamma _\text {POST}) \end{aligned}$$

for the projections in the dorsomedial loop and:

9 $$\begin{aligned} C_{ij} = (Tr_i-{\overline{Tr}}_\text {PRE}-\gamma _\text {PRE})^+({\overline{r}}_\text {POST}-r_j-\gamma _\text {POST}) \end{aligned}$$

for the projections in the dorsolateral loop.

Cortical projections to the subthalamic nucleus also follow equation 2 with $T_d=1$. Projections from the subthalamic nucleus to the pallidum follow equation 7 with $T_d=-1$.

A different set of dopaminergic cells projects to each loop. Each dopaminergic cell in the dorsomedial loop is associated with one of the six possible outcomes. In the dorsolateral loop, each dopaminergic cell is associated with one possible button. On both loops, dopaminergic cells project to all nuclei. Their activation is governed by the following equation:

10 $$\begin{aligned} \tau \frac{dm_j}{dt} + m_j = P(t)\Big (R(t)+Q(t)\sum _{i\in StrD1} w_{ij}r_{i}\Big ) + B_{DA} \end{aligned}$$

where *B* represents the baseline dopamine level at resting conditions. The function *P*(*t*) controls the timing of phasic changes. It returns 1 after every action and 0 otherwise. The function *R*(*t*) controls the size of the phasic changes and it is different in both loops. In the dorsomedial loop, *R*(*t*) is $(1-B)$ if the outcome associated with the cell is obtained and 0 otherwise. In the dorsolateral loop, *R*(*t*) is $(1-B)$ only if the button associated with the cell is pressed. Following the reward prediction error hypothesis, the response of dopaminergic cells is reduced through inhibitory plastic connections from the striatal D1 cells of the corresponding loop. Additionally, the function *Q*(*t*) is used to recreate the strong drop observed when a reward is predicted but not obtained. In the dorsomedial loop, $Q(t)=-10$ in unrewarded trials and $Q(t)=-1$ in rewarded trials.

Although dopamine-dependent plasticity is implemented in both loops, the respective dopamine signals differ and provide each loop with the appropriate prediction error information (Engelhard et al. 2019; Rusu and Pennartz 2020; Poulin et al. 2018). As in Baladron and Hamker (2020), dopamine in the dorsomedial loop encodes a classic reward prediction error signal while in the dorsolateral loop, it encodes an action consequence prediction error. Ongoing learning leads to a selective increase of the inhibitory projection from the striatum to the dopaminergic cells (prediction part) and thus reduces the error.

Successful trials increase the weights of the inhibitory connections from the striatal cells to the dopaminergic cells, following the equation:

11 $$\begin{aligned} \tau _{w}\cdot \frac{dw_{ij}(t)}{dt} = g_{DA}\cdot (DA(t)-B_{DA}) \cdot (r_{i}(t)-{\overline{r}}_\text {PRE})^{+} \end{aligned}$$

where $w_{ij}$ is the weight between the striatal cell *i* and the dopaminergic cell *j*,

12 $$\begin{aligned} g_{DA}= {\left\{ \begin{array}{ll} 1 &{} if\ reward\\ 3 &{} if\ no \ reward \end{array}\right. } \end{aligned}$$

for the dorsomedial cells and $g_{DA}=1$ for the dorsolateral cells, DA(t) is the sum of dopaminergic inputs to the striatal cell *i*, $r_i$ is the firing rate of striatal cell *i*, ${\overline{r}}_\text {PRE}$ is the mean of the presynaptic layer and $\tau _w$ is a time constant (3,000 ms for the dorsomedial loop and 12,000 ms for the dorsolateral loop).

Plasticity in the shortcut is not modulated by dopamine. These connections follow the correlation learning rule:

13 $$\begin{aligned} \begin{aligned} \tau _w\frac{dw_{ij}}{dt}&= C_{ij} - \alpha _j((r_j-{\overline{r}}_\text {POST}-\gamma _\text {POST})^+)^2w_{ij} \\ C_{ij}&= (r_i-{\overline{r}}_{PRE}-\gamma _\text {PRE})^+(r_j-{\overline{r}}_\text {POST}-\gamma _\text {POST}) \end{aligned} \end{aligned}$$

Further, these connections present slower changes than the cortico-striatal projections (controlled through different values of $\tau _w$). A pattern can only be obtained if the basal ganglia select the same action multiple times.

All differential equations are numerically solved using the Euler method with a time step of 1 ms using the neuro-simulator ANNarchy version 4.6 (Vitay et al. 2015).

### Task mapping

In order to simulate the task of Delorme et al. (2016) we included a set of 12 cells encoding the visual stimuli, 2 encoding each of the possible 6 fruit icons outside the boxes. These input cells had plastic connections to the dorsomedial loop (striatum and subthalamic nucleus) and to the shortcut (Fig. 2B).

An additional set of 30 input cells encoded a desire for a possible outcome fruit. Each of the 6 possible outcome fruits was therefore encoded by 5 cells. Fixed connections were included from these cells to the striatum and the STN of the dorsomedial loop, both of which were further divided into 6 groups of 4 neurons each, each receiving excitatory input from a different outcome fruit.

The cortex, GPi and GPe of each loop contain 2 cells each. In the dorsomedial loop these cells represent the possible keys that the subject may press. In the dorsolateral loop they represent the necessary hand movement to reach the buttons. Cortical cells in the dorsomedial loop receive an external input that increases their firing rate if their corresponding key was pressed at the end of a trial (independent of the selection done by the loop).

At the beginning of each simulated trial, the firing rate of the visual stimuli cells encoding one fruit was set to 0.5 and the firing rate of the cells encoding the corresponding outcome fruit was set to 1.0. Then, the network was simulated until either 600ms have elapsed or a cell in the cortex of the dorsolateral loop reached a threshold of 0.2. When the model did not reach the threshold, it was considered as if no response was produced. On trials in which the threshold was reached, the final decision was computed stochastically according to the categorical probability distribution obtained using the activity of the cortical cells in the dorsolateral loop:

14 $$\begin{aligned} P(a_i) = \frac{r_i}{\sum _i r_i} \end{aligned}$$

where $P(a_i)$ is the probability of selecting the action associated with the cortical cell *i* and $r_i$ is the firing rate of the cortical cell *i*.

After the decision, the phasic level of dopamine was changed according to equation 10 for an additional 100ms of simulation. The dopamine modulated learning rule was therefore only active during this period.

Finally, the input was removed and an inter-trial period of 700ms was simulated. This allowed the network to reach a stable initial condition before the next trial.

Each model was trained for 10 blocks in which each possible stimulus was presented twice. For each of the conditions described in the results section, 50 models were simulated, each with different initial conditions and noise values. After the training period, outcome devaluation and stimulus devaluation experiments were simulated. On outcome devaluation trials, no outcome encoding cell was activated. On stimulus devaluation trials, the firing rate of the corresponding visual input cells was set to a random value sampled from a normal distribution with mean 0.28 and standard deviation 0.05. This represents that the stimulus was still seen, but was unattended. The response rate in each case was computed as the number of trials in which the threshold was reached, divided by the total number.

### Pathological model alterations

Disturbed tonic dopamine signaling was simulated by multiplying the firing rate (*r* in Eq. 1) of the striatal D1 neurons by a factor bigger than 1 and the firing rate of striatal D2 neurons by a factor between 0 and 1. Disturbed phasic dopamine was simulated by decreasing the baseline value ($B_{DA}$). Such a change enhances phasic learning, as the plasticity rule depends on the difference between the current dopamine level and the baseline ($f_{DA}(DA(t) - B_{DA})$) and is only applied after the response. Reduced striatal inhibition was simulated by multiplying the weight of the local connection between striatal cells by different factors, as reported in the results section.

### Statistical analysis

Statistical significance between model groups was measured using a permutation test. On each test we first pooled both groups together and then re-sampled into two groups 1,000,000 times. For each sample, we computed the difference between the two groups and then used this result to estimate the *p*-value as the proportion of samples that had a difference greater or equal than the original value. If the estimated *p*-value was smaller than 0.005, the difference was considered significant.

## Declarations

### Code availability

The code to replicate all experiments is available from the corresponding author on request.
